# Environmental surveillance during an outbreak of tularaemia in hares, the Netherlands, 2015

**DOI:** 10.2807/1560-7917.ES.2017.22.35.30607

**Published:** 2017-08-31

**Authors:** Ingmar Janse, Miriam Maas, Jolianne M Rijks, Miriam Koene, Rozemarijn QJ van der Plaats, Marc Engelsma, Peter van der Tas, Marieta Braks, Arjan Stroo, Daan W Notermans, Maaike C de Vries, Frans Reubsaet, Ewout Fanoy, Corien Swaan, Marja JL Kik, Jooske IJzer, Ryanne I Jaarsma, Sip van Wieren, Ana Maria de Roda-Husman, Mark van Passel, Hendrik-Jan Roest, Joke van der Giessen

**Affiliations:** 1 Centre for Infectious Disease Control (CIb), National Institute for Public Health and the Environment (RIVM), Bilthoven, the Netherlands; 2 These authors share first authorship; 3 Dutch Wildlife Health Centre, Utrecht University, Utrecht, the Netherlands; 4 Department of Bacteriology and Epidemiology, Wageningen Bioveterinary Research (WBVR), Lelystad, the Netherlands,; 5 Department of Diagnostics and Crisis Organisation, Wageningen Bioveterinary Research (WBVR), Lelystad, the Netherlands; 6 GGD Fryslân, Regional Public Health Service of Friesland, Leeuwarden, the Netherlands; 7 Netherlands Food and Consumer Product Safety Authority, Wageningen, the Netherlands; 8 GGD Utrecht, Regional Public Health Service of Utrecht, Zeist, the Netherlands; 9 Resource Ecology Group, Department of Environmental Science, Wageningen University and Research Centre, Wageningen, the Netherlands; 10 Institute for Risk Assessment Sciences (IRAS), Faculty of Veterinary Medicine, Utrecht University, Utrecht, the Netherlands

**Keywords:** re-emerging diseases, environmental surveillance, zoonosis, outbreaks, Francisella tularensis, tularaemia

## Abstract

Tularaemia, a disease caused by the bacterium *Francisella tularensis*, is a re-emerging zoonosis in the Netherlands. After sporadic human and hare cases occurred in the period 2011 to 2014, a cluster of *F. tularensis*-infected hares was recognised in a region in the north of the Netherlands from February to May 2015. No human cases were identified, including after active case finding. Presence of *F. tularensis* was investigated in potential reservoirs and transmission routes, including common voles, arthropod vectors and surface waters. *F. tularensis* was not detected in common voles, mosquito larvae or adults, tabanids or ticks. However, the bacterium was detected in water and sediment samples collected in a limited geographical area where infected hares had also been found. These results demonstrate that water monitoring could provide valuable information regarding *F. tularensis* spread and persistence, and should be used in addition to disease surveillance in wildlife.

## Introduction

Tularaemia is a zoonosis caused by the intracellular pathogen *Francisella tularensis*. Disease in humans and animals is mostly caused by subspecies *tularensis* (type A) and *holarctica* (type B) [1]. In Europe, tularaemia is a locally emerging or re-emerging zoonosis with most human cases reported from Sweden, Finland and Turkey [1-4]. There may be sporadic and geographically confined cases, and seasonal epidemics and epizootics [5]. *F. tularensis* has a wide host range, although lagomorphs and rodents appear to be particularly susceptible to infection and symptom development [6]. Small mammals contribute to the geographical spread of *F. tularensis*, but they may not constitute the major reservoir [5]. Although tularaemia occurrence has been linked to particular landscape features [4,7], the dominant environmental reservoir(s) in which *F. tularensis* may persist for prolonged periods are still largely unknown and may vary between geographical areas [1]. *F. tularensis* has been detected in various types of surface waters and sediments [3,8] where it is potentially hosted by free-living protozoa [9]. Multiple transmission routes exist, resulting in different clinical manifestations. In humans, insect bites or direct contact with infected animals generally leads to ulceroglandular infections, ingestion of infected meat or water to oropharyngeal infections, and inhalation of infected aerosols to respiratory pneumonic infections [5].

In the Netherlands, indigenous tularaemia had not been reported for over 50 years, until a patient in the western province of Zuid-Holland was diagnosed with ulceroglandular tularaemia contracted from an unidentified local source in 2011 [10]. This first human case of a re-emerging disease initiated several actions. Medical and veterinary professionals were informed through a weekly surveillance report distributed by the National Institute for Public Health and the Environment (RIVM), and Dutch medical and veterinary journals [11-13]. After additional cases in the following years, a procedure to make tularaemia a notifiable disease in humans began, and was implemented in November 2016 [14]. Surveillance of brown hares (*Lepus europaeus*) started at national level in 2011 and through this, a hare infected with *F. tularensis* was identified in the southern province of Limburg in May 2013 [15]. Two months later, ulceroglandular tularaemia was diagnosed in a patient from the same region. In 2014, *F. tularensis* infections were detected in three humans (including two family members) and two hares. The human and hare tularaemia cases occurred in various geographical areas of the Netherlands and in different seasons [16].

### The event

On 9 February 2015, two hares were submitted to the Dutch Wildlife Health Centre (DWHC) for post-mortem investigation. The case history noted that four hares had died over the weekend in a garden in the province of Friesland, northern Netherlands, with death occurring abruptly after a spell of abnormal behaviour. The two animals were in fair condition and had acute severe extensive necrotic hepatitis. Tularaemia was confirmed by real-time PCR (qPCR). Following the two index cases, four more dead hares were found east and south of these two cases on 27 February, 4 March and 11 March. Tularaemia was confirmed to be the cause of death, and suspicion of a tularaemia outbreak was communicated in March. Following the event, active human case finding was initiated to reveal undiagnosed tularaemia cases. Potential reservoirs and transmission routes of *F. tularensis* in the area were investigated, including common voles (*Microtus arvalis*), arthropods and surface waters. Consideration of common vole involvement in the tularaemia outbreak among hares was based on an unusual population burst that had been observed in Friesland during the preceding winter of 2014/15, potentially providing a susceptible population for infection. Arthropods were studied to uncover their potential role in the transmission of *F. tularensis*.

## Methods

### Outbreak investigation

A zoonosis response team was organised by the RIVM to assess whether these findings implied a threat to public health. Short-term risks of *F. tularensis* infection due to skinning of animals or environmental exposure (e.g. inhalation of aerosols) were explored by surveillance of dead hares and surface waters. There were also additional investigations of voles and arthropods to address long-term risks since the persistence of stable *F. tularensis* reservoirs is particularly relevant in the summer when more people are exposed to surface water and arthropod bites.

### Collection and analysis of hares

In the Netherlands, passive surveillance of unusual mortality events in wildlife involves post mortem examination and diagnostic testing of dead animals submitted by volunteers. For brown hares, this includes histological examination at the DWHC and screening for *F. tularensis* infection of lung, liver and spleen tissue by qPCR-testing at Wageningen Bioveterinary Research (WBVR) [15]. Hares are mostly submitted by hunters, farmers and veterinarians. Following evidence of an outbreak among hares in Friesland in 2015, submitting dead hares from this province was actively encouraged by the DWHC. A convenience sample of 23 persons, mostly hunters from different game management units, were interviewed in June after the outbreak to gain more insight into the outbreak dynamics.

### Human case finding

The local public health service, GGD Fryslân, approached farmers and hunters who had handled infected hares to investigate whether these individuals had developed health problems. In collaboration with the regional laboratory for infectious diseases, Izore, patient records were retrospectively searched from the area of the hare epizootic in the months February, March and April 2015. The search included requests for laboratory diagnosis of pathogens causing clinical manifestations mimicking those of tularaemia (e.g. *Bartonella henselae*, cytomegalovirus, Epstein–Barr virus and *Toxoplasma gondii*), often by serology, but also including cultures of lymph node biopsies. General practitioners of 13 patients who met at least one of these criteria were contacted and the possibility of clinical tularaemia was reconsidered. Also, between February and December 2015, lymph nodes, mesenterium and neck biopsy samples were submitted to Izore for routine diagnostics from 18 patients whose symptoms were compatible with tularaemia. The samples were investigated for the presence of *F. tularensis* DNA using an in-house qPCR.

### Collection and analysis of common voles

Because the population burst of common voles caused great damage to grasslands, control measures were implemented in the winter of 2014/15. Common voles examined in this study originated from three of such control activities in 2015 (Table). One batch of 181 animals was captured using snap traps placed at different locations at the northern edge of the tularaemia epizootic between February and April (Table, Figure). A second batch of 38 animals was captured alive in Feytebuorren, west of the epizootic, in April (Table, Figure). The animals were handled in compliance with Dutch laws on animal handling and welfare (Dutch Animals Ethics Committee approval experiment 201400028). Whole blood samples were collected and spleens were harvested immediately after the animals were euthanised, flash frozen on dry-ice and stored at −80 °C. A third batch of 38 voles had been captured dead by farmers in Nes, at the centre of the epizootic, in August (Table, Figure). These specimens were stored at −20 °C until spleens were harvested. DNA from all specimens was isolated by using the DNeasy Blood and Tissue Kit (Qiagen, Hilden, Germany), and *F. tularensis* detection by qPCR was carried out at the WBVR [15] or RIVM [17]. The latter multiplex assay detects *F. tularensis* species based on multi-copy signature sequence IS*Ftu*2 and single-copy gene *fop*A, while gene *pdp*D is included to allow differentiation between subtype *holarctica* (gene absent) and other subtypes (gene present). In addition, the assay contains an internal control for assessing PCR inhibition. All qPCRs were run in triplicate.

**Table:** Tularaemia surveillance specimens and samples from potential reservoirs and transmission routes, the Netherlands, 2011–2016

Reservoirs, vectors		Collection dates	Collection locations^a^	Number of specimens/ samples^b ^analysed	Number of specimens/samples^b ^where *F. tularensis* DNA detected	Location on map (Figure)
Small mammals	Hares	Jul 2011–Dec 2014^c^	NL	106	3	NA
		Jan–Jun 2015	EZ	12	11	Large stars
			FL	15	1	
			NL	19	2	
		Jul–Dec 2015	EZ	7	0	Small stars
			FL	6	0	
			NL	34	0	
	Common voles	Feb–Apr 2015	FL, north of EZ	181	0	Triangles, ma
		Apr 2015	FL, west of EZ	38	0	Triangles, mb
		Aug 2015	EZ	38	0	Triangles, mc
Arthropods	Mosquito larvae	23, 29 Apr 2015	EZ, FL	124, pooled	0	Large diamonds
		28 Apr; 3, 12 May; 30 Jun 2016	EZ, FL	266	0	Small diamonds
	Mosquito adults	9, 10 Jul; 11, 14 Aug 2015	EZ, FL	371	0	
		28 Apr; 13, 15, 24, 30 Jun 2016	EZ, FL	296, pooled	0	
	Tabanids	3, 7, 11, 14 Aug 2015	EZ, FL	758	0	
		13, 30 Jun 2016	EZ, FL	6	0	
	Ticks	9, 10 Jul 2015	EZ, FL	220	0	
		28 Apr; 3 May; 13, 15 Jun 2016	EZ, FL	665, pooled	0	
Environment	Water	16 Apr 2015	EZ	7	6	Circles, A–D
			FL, north of EZ	1	0	Circle, E
		29 May 2015	EZ	27	8	Circles, A–T
			FL	5	0	Circles, U–X
		29 Jul; 10 Aug; 9 Sep 2015	EZ	12	1	Circles, B,D,M,R
	Sediment	16 Apr 2015	EZ	7	4	Circles, A­D
			FL, north of EZ	1	0	Circle, E
		29 May 2015	EZ	18	2	Circles, A–T
			FL	3	0	Circles, U–X

EZ: epizootic area within Friesland province; FL: Friesland province, but outside the epizootic area; NL: other parts of the Netherlands; NA: not applicable.

^a^ The epizootic area (EZ) was defined as the geographical area where several dead, tularaemia-confirmed hares were found in 2015, with the outer border being a distance of 5 km from any of these finding sites.

^b^ Numbers for animals refer to specimens, numbers for water and sediment refer to samples.

^c^ Hares collected before the event.

**Figure:**
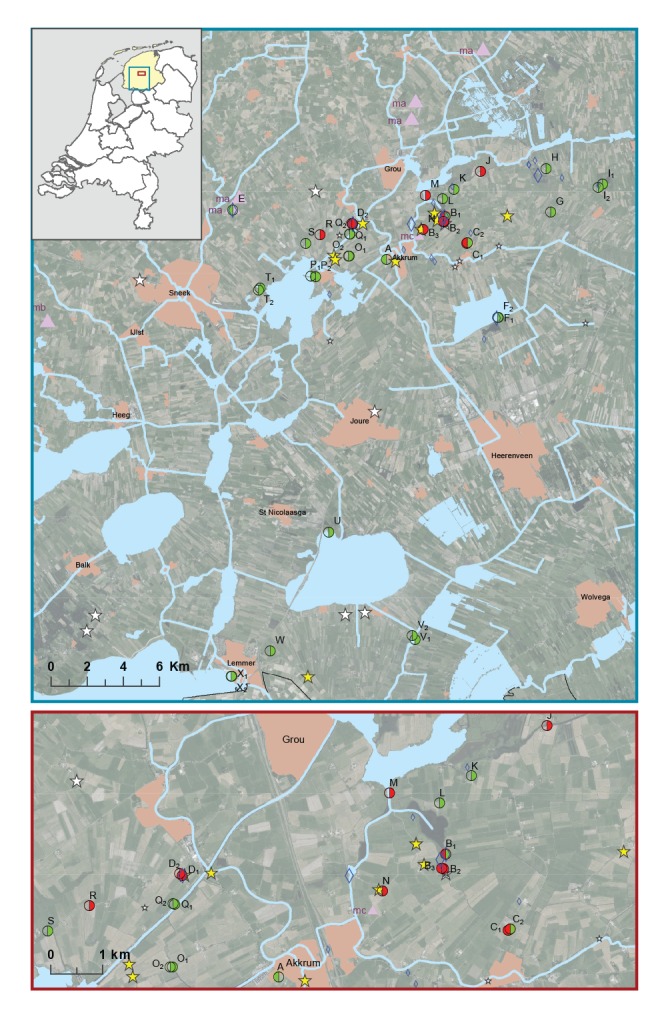
Locations of dead brown hares and other tularaemia surveillance samples collected during and after a tularaemia outbreak in hares, the Netherlands, 2015–2016

A–X: surface water sampling locations, numbers represent multiple sampling points at one location; ma: 181 common voles captured using snap traps between February and April; mb: 38 common voles captured alive in April; mc: 38 common voles. captured dead by farmers in August

The insert depicts a map of the Netherlands with the province of Friesland marked in yellow. The blue and red squares indicate the areas covered by the upper and lower maps, respectively. The lower map zooms in on the epizootic area. Locations where dead hares were found during the first and second half of 2015 are indicated by large and small stars, respectively. Yellow stars represent hares where tularaemia was confirmed as cause of death while white stars represent tularaemia-negative hares. Locations where common voles were collected during the first and second half of 2015 are indicated by large and small purple triangles, respectively. Locations where arthropods were collected during the first half of 2015 or in the second half of 2015 or later are indicated by large and small blue diamonds, respectively. Surface water sampling locations are represented by circles. Samples collected on 16 April and on 29 May are indicated by the left half and right half of the circle, respectively. Presence of *F. tularensis* DNA is indicated by the colours red (detected) or green (not detected). Transparency signifies that no samples were collected on that date.

### Collection and analysis of arthropod vectors

Selection of locations for arthropods collection was guided by finding sites of hares confirmed to have tularaemia and by *F. tularensis* DNA detected in water samples. Mosquito larvae were collected from suitable aquatic habitats in April 2015 and in April, May and June 2016. In July and August 2015, and in April, May and June 2016, adult mosquitos, tabanids and ticks were collected using BG sentinel traps, manning traps and dragging cloth method, respectively (Table, Figure). DNA extraction from individual ticks was carried out using alkaline lysis and DNA extraction from individual or pooled tabanids and mosquitos using the DNeasy 96 Blood and Tissue Kit (Qiagen). *F. tularensis* detection was performed by qPCR [17].

### Collection and analysis of surface waters and sediments

Samples from surface waters and sediments were collected from the geographical region where the infected hares had been found. On 16 April 2015, five ditches separated by at least 1.5 km were sampled (Figure, locations A to E). At two of these locations, samples were collected from more than one sampling point (indicated by a number on the map). Sampling locations A to D were based on reported finding sites of dead hares confirmed to have tularaemia that were submitted between February and April. Locations B and C were further guided by where farmers indicated there was high local hare mortality. Location E had no findings of dead hares, but it was included because of a large local population increase of common voles. On 29 May, water samples were collected from 32 sampling points at 22 locations (Figure, locations B to D and F to X). Sampling points included 20 ditches similar to the first sampling, but also covered six sites at the banks of canals and six at the shores of lakes. Five sampling points (P_1_, T_1_, U, V_1_, X) are official swimming locales, while some of the other sampling points (I_1_, J, K, M) are also used for swimming and recreation. At four sampling points (B_2_, D_1_, M and R), additional water samples were collected by the regional water authority of Friesland, Wetterskip Fryslân, in July, August and September 2015.

Water samples of 1 L and sediment samples were transported to the RIVM where the water samples were filtered using membranes with a pore size of 0.45 μm until they clogged. Filters and sediment slurries were stored at −20 °C until DNA was extracted by using the PowerWater and PowerSoil DNA extraction kits (Qiagen, Hilden, Germany), respectively. *F. tularensis* DNA was detected by using qPCR [17]. Primers from this assay were used to produce amplicons for sequencing analysis.

## Results

### Outbreak investigation

Overall, 40 hares were submitted to the DWHC from the province of Friesland in 2015, compared with two or less per year in the period 2011 to 2014. Tularaemia infection was confirmed in 12 of these 40 hares, with 11 of the cases occurring in a geographically limited area of ca 50 km^2^ (Table, Figure). The last confirmed case was found on 15 May. Hunters from the area of the epizootic estimated that by June 2015, the hare population had decreased by at least two thirds, while hunters from other areas reported less or no declines. For some hunting grounds in the area, hunters also reported unusually high hare numbers before the outbreak. Outside Friesland, 2 of 53 submitted hare specimens in 2015 were infected with *F. tularensis* (Table), consistent with the sporadic tularaemia cases in hares observed since 2013.

### Human case finding

There were no reports of human tularaemia patients in Friesland in 2015. Active case finding approaches, including searching relevant patient records and PCR testing of diagnostic materials from patients with compatible symptoms, did not reveal any missed tularaemia cases.

### Investigation of common voles and arthropods

A total of 257 common voles were collected from different locations in the vicinity of the hare epizootic between February and August 2015 (Figure) and all tested negative for *F. tularensis* infection. Similarly, *F. tularensis* DNA was not detected in mosquito larvae (n = 390), adult mosquitos (n = 667), tabanids (n = 764) and ticks (n = 885), collected in the area of the epizootic (Table).

### Investigation of surface waters and sediments

*F. tularensis* DNA was detected in water samples collected on 16 April 2015 from three of four locations in the geographical region where the infected hares had been found (Figure, locations B, C and D). All sampling points at locations B and C showed positive results. All three replicates were positive for the multi-copy signature sequence IS*Ftu*2, whereas the *fop*A gene was not always detected in each replicate, indicating that its concentration was around the limit of detection (LOD). The *pdpD* gene was never detected. At location D, *F. tularensis* DNA levels were higher compared with the other locations. This was indicated by relatively low qPCR threshold (Cq) values (e.g. 27.5 for signature sequence IS*Ftu*2, which was 5–8 Cq lower compared with the other locations) and consistent *fop*A gene detection in all replicates. *Fop*A genes could be amplified and sequenced from locations B and D, showing 100% similarity to published *F. tularensis* sequences. In sediment samples, *F. tularensis* DNA was detected at all four sampling points from two locations (B_1_, B_2_, B_3_, D_1_) that had tested positive for *F. tularensis* DNA in water, albeit at higher Cq values compared with the corresponding water samples.

Based on these findings, additional samples were collected to investigate possible persistence of *F. tularensis* at the contaminated locations, as well as to explore its occurrence in a wider area. Of the follow-up water samples collected on 29 May 2015, *F. tularensis* was again detected at locations B and D, but not at location C. It was also detected at four additional locations: J, M, N and R (Figure). These locations, which included ditches and canals, were all in close proximity to the locations recognised to be contaminated from the earlier sampling. Sediment samples were collected at 21 of the 32 water sampling points, and *F. tularensis* DNA was detected only at two sampling points, one from location B and one from location D. The amount of *F. tularensis* DNA in the positive samples appeared to be lower when compared with the first measurements given the relatively high Cq values and occasional negative replicates for the multi-copy target *ISFtu2*, and that the *fop*A gene was only detected in one instance. In July, August and September 2015, water sampling was repeated at locations B, D, M and R, but *F. tularensis* DNA was only detected at location D in July (Table).

## Discussion

Sporadic tularaemia cases, i.e. cases that do not cluster by space or time, in humans and in hares in the Netherlands [10-13,15,16] suggest widespread occurrence of *F. tularensis* and the existence of an endemic cycle of the pathogen. Its ability to cause epizootics was uncovered by the sharp increase in the number of dead hares reported from a geographically restricted region in Friesland, with > 90% of carcasses submitted from this region tested positive for tularaemia (Table). Although the size of the hare population was not investigated systematically, anecdotal reports from farmers and hunters pointed to an obvious decline in the local hare population, supporting the notion of a tularaemia epizootic. The start of the epizootic may have been exacerbated by high hare density in some areas as well as by frequent and close contacts among hares during courtship (boxing) which normally peaks between February and April. A hare epizootic in France in 2011 also occurred during mating season [18]. However, the temperature drop preceding hare mortality during that outbreak was not observed in Friesland; instead, the preceding winter had been relatively mild. Although direct contact between hares was probably responsible for spreading of tularaemia during the epizootic in Friesland, sporadic *F. tularensis* infections in dispersed regions throughout the Netherlands points to the existence of more stable environmental reservoirs.

The low tularaemia incidence in the Netherlands [10-13,15,16] could be due to a patchy distribution of such reservoirs, or to incidental exposure to susceptible hosts. Small mammals, particularly rodents, are highly susceptible to infection and could be exposed to the same environmental reservoirs as hares [5,6,19]. Common vole population dynamics have been suggested to be a major driving force of tularaemia ecology, including determining tularemia incidence in hares and humans [19,20]. However, we did not detect common voles infected with *F. tularensis* during their population burst and thus found no support for a tularaemia epizootic similar to that among hares. Blood-feeding arthropods have been recognised as potential mechanical (tabanids, mosquitoes) and biological (ticks) transmission vectors [21], but we could not substantiate their role in tularaemia transmission. The results of this study may suggest a limited role for common voles and arthropods in tularaemia ecology, yet the absence of infected animals could also be explained by: (i) a mismatch between the time points and locations from which animals were collected and the hare epizootic, and (ii) prevalence of infected animals below the detection limit. An additional explanation for not detecting infected voles could be (iii) that infected animals died rapidly in inaccessible locations, like burrows.

We detected *F. tularensis* DNA both in surface waters and sediments at different locations in the area of the hare epizootic, which supports persistence of the bacterium in aquatic environments in the Netherlands. The distribution of locations positive for *F. tularensis* in Friesland suggests that environmental conditions are favourable for growth of *F. tularensis* to detectable levels only at some locations; all locations positive for *F. tularensis* were restricted to a limited geographical area associated with hare mortality of roughly 50 km^2^. Locations where higher concentrations of *F. tularensis* DNA were detected (B and D) corresponded to the geographical centre of the epizootic; the first reported dead hares were found near location D. Although the number of sampling points was insufficient for inferring preferential habitat features, there seemed to be a bias towards small, shallow ditches when compared with larger canals or lakes. A temporal dynamic of *F. tularensis* in surface waters was indicated by its disappearance from locations B, D, M and R after the hare epizootic had ended. Environmental persistence, independent of a vertebrate host, and infectivity of *F. tularensis* will depend on local factors such as salinity and temperature, and may be linked to its ability to form biofilms and reside in protozoa hosts [22,23]. An epizootic may start with an infection from a local aquatic reservoir where *F. tularensis* persists. However, at least some of the *F. tularensis* DNA detected in surface waters may also originate from transient contamination by infected hares. Dead hares were sometimes found at or near banks. A mechanism where animals with a high bacterial load act as amplifiers contaminating the local environment was considered in other reports of *F. tularensis* DNA in aquatic samples [8,24]. Nevertheless, the persistence of *F. tularensis* in water for several years and between outbreaks as found by Broman et al*.* [8] supports the existence of more stable aquatic reservoirs.

The concurrence of the epizootic and *F. tularensis* DNA in water suggested a link between infection of hares and aquatic reservoirs of subspecies *F. tularensis holarctica*. However, low DNA concentrations impeded subtyping to confirm such a link and the presence of closely-related subspecies, such as *F. novicida,* in some of the environmental samples cannot be ruled out [25,26].

*F. tularensis* DNA was detected in six sediment samples, from 12 different sampling points where water samples were contaminated and sediment samples were available. From these data it cannot be concluded whether this is because of lower *F. tularensis* abundances in sediments or methodological differences since analysed volumes and extraction methods differed between sediment and surface water analyses. Broman et al. [8] also observed a lower number of positive samples in sediments, and sediment and water sample volumes in their study were more similar. In their study, *F. tularensis* was detected in surface water samples of only 2 mL. Their detection limit was estimated to be 10^3^ bacteria per mL natural water sample. We filtered 100 to 600 mL of water, depending on the water turbidity, which resulted in a detection limit of less than 1 genomic equivalent (GE) per mL of natural water [17]. These sensitivities cannot be directly compared, mostly because the LOD calculated by Broman et al. was based on spiked cultivated bacteria and not purified genomic DNA, and the latter method does not consider losses due to filtration and DNA extraction. Nevertheless, the differences are considerable and the inclusion of a filtration concentration step would make the method we used more sensitive. Since the concentrations of *F. tularensis* in our study were just above the detection level, it is likely that the concentrations in the Swedish waters that Broman et al. investigated were higher than those in Friesland.

Recent findings of surface waters positive for *F. tularensis* at locations in central and south-western the Netherlands that were associated with hare or human tularaemia cases, support the notion that monitoring surface waters may help signal potential public health threats and be used to better understand the environmental component of this zoonosis. Water monitoring has also been successfully used for the detection of opportunistic pathogens with an environmental component, such as *Burkholderia pseudomallei* [27] or *Vibrio* spp [28].

During the hare epizootic in 2015 we found no human cases, even after active case finding efforts. Human infections may have been prevented by local health authorities’ active dissemination of advice to not skin and consume hares, combined with the epizootic starting after closing of the hunting season on 15 January. Current human tularaemia cases in Europe are linked to the skinning of animals, arthropod bites, or contaminated drinking water or dust [1,3,29]. Of the six human tularaemia cases identified in the Netherlands between 2011 and 2015, three were linked to skinning of hares and one to insect bites; two cases may have resulted from exposure to an environmental source [11-13,16]. Health risks from exposure to *F. tularensis* in surface water are more difficult to assess and control, but cannot be excluded. An epidemiological description of tularaemia in Sweden illustrated that disease incidence is highest near lakes and rivers [7]. Also, the occurrence of *F. tularensis* in surface water and sediment has been associated with human tularaemia outbreaks [8]. Waterborne infections may occur after ingestion of water, inhalation of aerosols or entry through skin, including while fishing [30]. An alternative theory on water-transmitted tularaemia involves blood-feeding mosquitos taking up bacteria during their aquatic larval stage, but there is no direct evidence for such transmission, including by a trans-stadial route [31-33]. During the epizootic in Friesland, health risks from *F. tularensis* exposure through ingestion or skin contact were probably low as the bacterium was mostly detected before the recreational season and predominantly in small ditches. Crayfish fishing, which poses a potential transmission route, is very uncommon in the Netherlands [30]. On the other hand, at least two contaminated locations (J an M) are associated with recreational activities (sailing and swimming). A potential health risk could arise from inhaling contaminated aerosols when surface waters are used for spray irrigation on farms, which is of particular concern because inhalation of *F. tularensis* is associated with the most serious form of tularaemia [34].

## Conclusion

Input from different types of surveillance is required to signal potential local or seasonal hazards, including hazards from the introduction of emerging and re-emerging pathogens. In addition to its role in signalling potential public health threats, surveillance data can be used to better understand the environmental components that may cause observed changes in the abundance of pathogenic microbes. This study shows that for tularaemia, valuable information regarding the spread and persistence of its causative agent, *F. tularensis,* could be derived from water monitoring in addition to disease surveillance in wildlife. Water data can be obtained relatively easily, but more extensive monitoring is necessary to elucidate the significance of detectable levels of *F. tularensis* in surface waters in terms of human and animal infection risks.
